# Phase I/II Study of Extreme Hypofractionated Stereotactic Body Radiation Therapy Boost to Prostate for Locally Advanced, Node-Positive and Oligometastatic Cancer

**DOI:** 10.7759/cureus.11751

**Published:** 2020-11-28

**Authors:** Kushal Narang, Mohit Kadian, K Venkatesan, Saumyaranjan Mishra, Shyam Bisht, Deepak Gupta, Susovan Banerjee, Tejinder Kataria

**Affiliations:** 1 Division of Radiation Oncology, Medanta Cancer Institute, Medanta The Medicity, Gurugram, IND

**Keywords:** advanced, extreme hypofractionation, node-positive, oligometastatic, prostate, boost, sbrt

## Abstract

Introduction: Stereotactic body radiation therapy (SBRT) is increasingly being utilized to deliver escalated radiation doses for improving outcomes in various malignancies. We analyzed our cohort of locally advanced, node-positive, and bone oligometastatic prostate cancer patients, that were treated with a combination of pelvic RT using conventional fractionation (CF) and SBRT boost to prostate using extreme hypofractionation (EH), along with hormone therapy (HT).

Materials and Methods: Outcomes of 44 prospectively treated patients were analyzed. Volumetric modulated arc therapy (VMAT) was utilized to deliver a dose of 45 Gy to pelvic nodal region, 50 Gy to prostate, and 54-56 Gy to gross nodes in 25 fractions. EH boost 18 Gy in three fractions was delivered to the prostate using CyberKnife (Accuray, Sunnyvale, CA, USA) SBRT. Bone oligometastasis, if any, were treated to a dose of 16 Gy in two fractions, delivered on weekends. Serum prostate-specific antigen (PSA), multi-parametric magnetic resonance imaging (MRI) of pelvis, and prostate-specific membrane antigen-positron emission tomography (PSMA-PET) were used for response assessment during follow-up. HT was given as per standard guidelines.

Results: There were 33 (75%) locally advanced, nine (20.5%) node-positive, and two (4.5%) oligometastatic cases. At a median follow-up of 63.5 months, the five-year progression-free survival (PFS) was 88.2%, biochemical PFS (bPFS) was 91.4% and overall survival (OS) was 96.9%. Grade III or greater acute genitourinary and gastrointestinal toxicity was 2.3% each, and late toxicity was 4.5% and 0%, respectively.

Conclusion: Excellent five-year outcomes can be attained even for locally advanced, node-positive and bone oligometastatic prostate cancer, by means of dose-escalation using EH-SBRT boost to the prostate.

## Introduction

Prostate cancer (PC) has an alpha/beta (α/β) value of around 1.5, which is lower than that of nearby critical structures like the bladder and rectum. This means that irradiating with doses per fraction greater than conventional fractionation (CF) would deliver a greater biologically equivalent dose (BED) to the prostate, without significantly increasing late normal tissue toxicity. The outcomes of studies employing dose-escalated CF (78-81 Gy) [1] are comparable with those of moderate hypofractionation (MH; 2.4-4 Gy per fraction) [2-3]. In recent years, multiple phase two and phase three studies employing extreme hypofractionation (EH; ≥5 Gy per fraction) have also reported excellent control rates with acceptable bladder and rectal toxicity. Although most of these studies have evaluated low-intermediate risk, localized PC [4], data for EH for high-risk, localized and advanced PC is rapidly accumulating [5-12]. However, use of this radical approach for oligometastatic cases is rarely reported. Since September 2012, we have been treating locally advanced, node-positive and bone oligometastatic PC cases with a combination of CF for prostate and pelvic nodal volume, with an EH boost to prostate and weekly hypofractionated radiation therapy (RT) for the metastatic site. We hereby report the five-year outcomes for this cohort.

## Materials and methods

We reviewed our prospectively maintained dataset of consecutively treated PC cases. From this dataset, patients with locally advanced and bone oligometastatic PC, treated with a combination of CF using volumetric modulated arc therapy (VMAT) and EH boost to prostate using CyberKnife® (CK; Accuray, Sunnyvale, CA, USA) stereotactic body radiation therapy (SBRT), were identified. Patients with less than one year of available follow-up were excluded. Thus, 44 patients, treated between August 2012 and June 2019, constituted the study dataset.

Treatment methodology

All patients underwent complete baseline staging work-up, including clinical examination, serum prostate-specific antigen (PSA), multi-parametric magnetic resonance imaging (MRI) of the pelvis including MR spectroscopy, diffusion-weighted imaging (DWI) with absolute diffusion coefficient (ADC) mapping, 12-core biopsy of the prostate including Gleason’s Score (GS) and a bone scan. Prostate-specific membrane antigen positron emission tomography (PSMA-PET) scan was available after September 2015. Staging and risk stratification was done as per National Comprehensive Cancer Network (NCCN) guidelines [13]. Bone oligo-metastasis was defined as presence of up to five metastatic sites. Treatment was initiated with neoadjuvant HT using bicalutamide and a luteinising hormone releasing hormone agonist (combined androgen block). After eight to 12 weeks, digital rectal examination, serum PSA and MRI pelvis were done to assess the prostate volume and prostate-rectum interface. Uroflowmetry and assessment of International Prostate Symptom Score (IPSS) were done for all patients. SBRT with EH was considered for patients with prostate volume <50cc and a distinct prostate-rectum interface. Contraindications for use of EH were prior history of trans-urethral resection of prostate (TURP), and significant urinary symptoms (IPSS ≥15).

RT planning and delivery

Preparation, Mould Procedure and Planning Scans

Treatment by CK-SBRT necessitated prior placement of three to four gold seed fiducial markers in the prostate for intrafraction motion tracking. After ensuring normal bleeding and clotting parameters, and under prophylactic antibiotic cover, fiducial markers of 3-5mm length and 1mm diameter were placed at the apex, base and lateral edges of the prostate using trans-rectal ultrasound guidance, and their positional geometry verified by X-ray imaging on CK. The patients underwent planning scans after five days to allow for fiducial stabilization and settlement of post-procedure oedema. Patients consumed two tablets of activated charcoal every night, starting two days prior to the date of RT planning, as part of bowel preparation. On the day of planning, patients were immobilized in supine position with their arms overhead in a vacuum cushion molded to match their body contours. They were kept nil by mouth for four hours prior, and rectal emptying ensured by administering an enema one hour prior to the scan. The volume of interest was selected from first lumbar vertebra to mid-thigh region, keeping a minimum 10-15cm margin above and below the target region. A non-contrast scan with 1mm slice thickness with empty bladder was performed for CK-SBRT. Subsequently, three external fiducial markers were placed over the pelvis using alignment lasers and a 3mm contrast-enhanced scan with mid-filled bladder was performed for the fractionated RT phase.

Target Delineation

Firstly, accessory imaging including MRI and PSMA-PET were fused with the planning CT scan image-sets. The prostate, any associated extracapsular or seminal vesicle (SV) involvement, gross nodes and oligometastatic bone lesions were marked as respective gross target volumes (GTV). The uninvolved part of seminal vesicles was not included in GTV. For VMAT utilizing daily inter-fraction imaging, a planning target volume (PTV) of 10mm all around, except for 5mm in posterior direction was added to the prostate GTV. These margins helped encompass the major part of seminal vesicles, and part of the bladder to account for minor filling uncertainties. For CK-SBRT with intrafraction imaging, a 3-5mm PTV was grown all around the GTV prostate, except for 2-3mm in posterior direction, taking care to be off the rectal wall. The pelvic nodal clinical target volume (CTV) was demarcated as per Radiation Therapy Oncology Group (RTOG) guidelines [14]. A 5mm three-dimensional margin was given around the gross nodes and the pelvic nodal CTV to generate the respective nodal PTVs. The bladder, rectum, bowel, femoral heads, pelvic bones and penile bulb were contoured as critical structures. The urethra was contoured on MRI, as described previously [15]. The dose prescription for VMAT in 25 fractions was 45 Gy to pelvic nodal PTV, 50 Gy to prostate PTV, and 54-56 Gy to gross nodal PTV; to be treated as five fractions per week. Bone oligometastases, if any, were treated with 16 Gy in two fractions, delivered on weekends. The dose prescription for CK-SBRT was 18 Gy in three fractions, prescribed to the 75-80% isodose covering the margin of prostate PTV. The dose-constraints for critical structures are mentioned in Table 1.

**Table 1:** Dose constraints for planning PTV: Planning Target Volume, EH: Extreme Hypofractionation, CF: Conventional Fractionation, Dmax: Maximum dose, Dmean: Mean Dose D195cc: Dose to 195 cc of the structure, and so on D20: Dose to 20% volume of the structure, and so on

Organ at Risk	Dose Constraint for EH phase (18 Gy)	Dose Constraint for CF phase (45-56 Gy)
Rectum	Dmax ≤ 18 Gy	Dmax ≤ 50 Gy
	D20 ≤ 12 Gy	D20 ≤ 35-40 Gy
	D10 ≤ 15 Gy	D30 ≤ 30-35 Gy
		D50 ≤ 25-30 Gy
Rectum - PTV	Dmax ≤ 18 Gy	Dmax ≤ 45 Gy
	D20 ≤ 10 Gy	D20 ≤ 30-35 Gy
	D10 ≤ 12 Gy	D30 ≤ 25-30 Gy
		D50 ≤ 20-25 Gy
Bladder	Dmax ≤ 18 Gy	Dmax ≤ 50 Gy
	D20 ≤ 12 Gy	D20 ≤ 40-45 Gy
	D10 ≤ 15 Gy	D30 ≤ 35-40 Gy
		D50 ≤ 25-30 Gy
Bladder - PTV	Dmax ≤ 18 Gy	Dmax ≤ 45 Gy
	D20 ≤ 10 Gy	D20 ≤ 35-40 Gy
	D10 ≤ 14-16 Gy	D30 ≤ 30-35 Gy
		D50 ≤ 20-25 Gy
Bowel	Dmax ≤ 10-15 Gy	Dmax ≤ 45-50 Gy
		D195cc ≤ 30-35 Gy
Penile Bulb	D2cc ≤ 15 Gy	Dmax ≤ 50 Gy
		Dmean ≤ 30 Gy
Urethra	Dmax ≤ 18 Gy	Dmax ≤ 50 Gy
Femur Heads	D10 ≤ 6 Gy	D50 ≤ 20 Gy
Pelvic Bones	D20 ≤ 8-10 Gy	D50 ≤ 20 Gy
Genitalia	D10 ≤ 4 Gy	Dmean ≤ 5 Gy
Thecal sac		Dmax ≤ 30 Gy

Treatment Plan Generation and Evaluation

VMAT planning was carried out on Elekta Monaco® (Elekta, Stockholm, Sweden) treatment planning system, version 5.1.1. Two-stage optimization was performed using inverse planning. The constrained and Pareto optimization algorithm performed the gradient search for the most feasible solution to achieve acceptable target coverage with optimal organ sparing, followed by Monte Carlo algorithm for final dose calculation using 2mm grid size. Two complete arcs (360 degrees), with arc length starting from 180 degrees to -180 degrees were most commonly used. For PTV, the goal was to deliver >98% of the prescribed dose to >95% of the volume, while maintaining maximum possible dose homogeneity, and respecting normal tissue constraints. Plan specific quality assurance was performed to verify parameters like gantry angle, multi-leaf collimator (MLC) shape and dose rate before treatment delivery for each patient. CK-SBRT planning was carried out on Accuray MultiPlan® treatment planning system, version 4.6.0. The initial step was tracking the implanted fiducials to the tracking synchrony of target localization. A minimum of three fiducials’ definition was necessary for correlating the translational and rotational prostate variation during beam delivery. Inter-fiducial distance of >2cm and angulation >15° was ideal for achieving optimal correction strategy. Treatment anatomy of body and prostate path set were chosen to define the robot movement around the patient surface in the predetermined cloud space, with each robot position on this cloud being the designated “node”. Suitable collimator sizes were chosen as per the target dimension. Limitation of monitor units (MU) for each beam and each node was set to achieve beam stability and correction of setup errors. Eight conformity shells were created surrounding the target to achieve target dose conformity and reduction of normal tissue doses. The gradient search sequential optimization algorithm then performed the dose optimization based on the dose constraints and objectives. After achieving an optimal plan, treatment time reduction was performed by optimizing robot movement, and beam reduction was done to reduce the lesser MU beams that were causing beam delivery instability. Subsequently, the final dose calculation was performed by using the ray tracing algorithm with a grid size of 1mm. The goal was to cover >98% of the prostate PTV with the prescribed dose, while concentrating hot-spots within the prostate GTV. Care was taken to avoid hot-spots over the urethra and to reduce the irradiated volume of the penile bulb, rectal wall, and bladder neck, while respecting all other organ constraints. After plan approval, quality assurance was performed in phantom for that specific plan to verify all parameters like robot position, MU delivery, and dose accuracy before proceeding for treatment delivery. The bone oligometastatic site was treated with SBRT or less conformal techniques as deemed feasible.

Treatment Delivery

In the initial years, the CK-SBRT phase utilizing EH succeeded the VMAT based CF phase by approximately two weeks. An internal audit showed difficulties in delineating the outer surface of the prostate, and also greater fiducial tracking uncertainties with this approach. The former could probably be due to radiation-induced inflammation and the latter due to a greater time-gap between fiducial placement and treatment on CK. Hence, we changed our practice to treat the CK-SBRT phase first, followed by VMAT in the following week (reverse-boost approach). For CK-SBRT, patients were treated on empty bladder to maintain the geometry of implanted fiducials for proper tracking and to avoid discomfort to the patient during the long treatment fraction. Intrafraction imaging helped identify and correct translational and rotational displacements in real-time. The CK system utilizes a 6MV Flattening-Filter-Free (FFF) beam with a dose rate of 1000 MU/min. Treatment fractions were delivered on an alternate-day basis, with each fraction lasting about 30-40 minutes. For VMAT, all patients were treated with dedicated bladder filling and rectal emptying protocol with daily bowel preparation, under stringent daily image guidance on Elekta Infinity® using X-ray volumetric imaging (XVI). Translational and rotational errors were corrected by means of Hexapod couch. A 6MV beam utilizing a dose rate of 200-400 MU/min was used. Each treatment fraction typically lasted for about seven to 10 minutes. Patients were reviewed daily during RT for ensuring appropriate execution of treatment delivery as per institutional standards and for recording and treating acute toxicity.

Follow-up evaluation

After RT completion, patients were followed-up initially after four to six weeks, and subsequently after every three to four months for the first two years, followed by every six months for the next three years, and annually thereafter. Serum PSA was done three-monthly for two years, and six-monthly thereafter. MRI pelvis or PSMA-PET were performed at six months post-RT, and annually thereafter. HT was continued for at least three years, or till a sustained PSA level of 0.02 ng/ml was attained across three successive six-monthly readings, whichever was later. Patients were prescribed bisphosphonate preparations to maintain bone health while on HT, and bone mineral density was checked annually. Acute and late toxicity was recorded as per Common Toxicity Criteria for Adverse Events, version 4.0 [16]. Progression was defined as local, regional or distant progression of disease on clinical examination or imaging, after due confirmation by cytology or biopsy from any of the progressed site(s). A biochemical progression was deemed to have occurred in case the PSA increased 2ng/ml above the nadir PSA (nPSA + 2ng/ml) in accordance with the Phoenix modification of ASTRO definition [17]. The progressed disease was appropriately managed by either changing or restarting HT, switching to chemotherapy, or palliative radiation therapy.

Statistical analysis

Data tabulation and basic statistics were performed using Microsoft Excel® 2013. The progression-free survival (PFS), biochemical progression free survival (bPFS), and overall survival (OS) were estimated through Kaplan-Meier survival statistics using MedCalc® software, version 19.5.2.

## Results

Patient characteristics

Median age was 69 years (range 52-84 years) and median initial PSA (iPSA) was 26ng/ml (range 4.8-217ng/ml). Thirty-two (73%) patients had GS 7 or 8. There were 33/44 (75%) locally advanced (T3a/bT4 N0), 9/44 (20.5%) node-positive (N1), and 2/44 (4.5%) bone oligometastatic (M1) cases. One oligometastatic patient had a single D7 vertebral lesion and the other had two lesions in the left iliac bone. Patient characteristics are summarized in Table 2.

**Table 2:** Patient Characteristics GS: Gleason's Score, iPSA: initial PSA, RT: Radiation Therapy

Parameter	Value (Range)
Age (years)	Median = 69 (52 - 84)
GS	Median = 8 (6-9)
6	4
7	17
8	15
9	8
iPSA (ng/ml)	Median = 26 (4.8 – 217)
Stage / Risk Group	Majority T3b / Very High
T3a / High	11
T3b / Very High	20
T4a / Very High	2
N1 / Regional	9
M1 / Metastatic	2
Prostate Volume at baseline	Median = 45.9 cc (23 – 104.8 cc)
Prostate Volume before RT	Median = 23.5 cc (12.7 – 68.5 cc)

Median follow-up, as calculated from initiation of neoadjuvant HT, was 63.5 months (range 17-92 months) and median HT duration was 31.5 months (range eight to 80 months), with the overall HT compliance in terms of taking HT till the prescribed duration being 86.4% (38/44 patients). The median duration of neoadjuvant HT was three months (range one to 10 months). The planning dose-constraints were achieved in all patients, and all patients could complete the full course of pelvic and prostate RT. The initial 15 (34%) patients received EH boost after the CF course, and the remaining 29 (66%) patients received it prior (reverse-boost approach).

Outcomes

**Figure 1:**
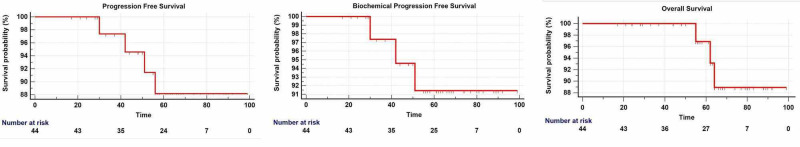
Progression-Free Survival, Biochemical Progression-Free Survival and Overall Survival

The median nPSA was 0.01ng/ml (range 0.002 - 0.8ng/ml), and the median time to reach nPSA was 13.5 months post-RT (range three to 63 months). Disease progression was noted in four patients, of which two had local and two had distant progression. Both patients experiencing local progression belonged to the Very-High risk category (T3b/T4a, N0M0). Of the patients experiencing distant progression, one patient belonged to the Very-High risk category (T3bN0M0) and one was oligometastatic (T3bN1M1) at baseline. The treated oligometastatic bone lesions were controlled on long-term follow-up. The five-year PFS was 88.2%, bPFS was 91.4% and OS was 96.9% (Figure 1).

Overall, mortality occurred in three patients, all from causes not related to PC. Mortality in two of the patients occurred after the five-year time point, and hence the six-year OS dropped to 89%. Incidence of acute genito-urinary (GU) and gastrointestinal (GI) toxicity was 2.3% each. One patient developed grade 3 bladder outlet obstruction, requiring channel TURP within three months post-RT. One patient had per rectal bleed from internal haemorrhoids for which RT had to be temporarily withheld and the bleed was managed with Argon Laser photocoagulation. Two patients developed urethral strictures requiring dilatation at more than two years post-RT (Grade III late GU toxicity = 4.5%). No patient developed Grade III late GI toxicity. HT related toxicity included gynaecomastia, andropausal behavioral symptoms, and osteoporotic vertebral collapse in one patient each.

## Discussion

This series is unique in that it exclusively consists of loco-regionally advanced and oligometastatic PC, unlike previously published studies of EH boost or of EH alone that report outcomes of localized and advanced PC together. Secondly, the oligometastatic cases in our study were treated with a more radical intent and received the same treatment as non-metastatic cases. The prostate (α/β = 1.5) received a BED of 206.7 Gy, which translates into a 2-Gy Equivalent (EQD2) of 88.6 Gy. The gross nodes (α/β = 1.5) received BED between 131.8 to 139.6 Gy (EQD2 56.5 to 59.8 Gy) and the nodal region (α/β = 1.5) received BED 99 Gy (EQD2 43.2 Gy). Bladder and rectum (α/β = 3) received a maximum BED of 137.3 Gy, with an EQD2 of 82.4 Gy, and bowel (α/β = 3) received maximum BED of 72 Gy (EQD2 43.2 Gy).

Zaorsky et al., in their meta-analysis of conventional and hypofractionated schedules [18], demonstrated that BED of around 200 Gy to prostate is important for attaining favourable long-term control rates, and restricting critical structure BED around 133 Gy helps limit normal tissue toxicity. While this is easier for capsule-confined localized PC, dose-escalation for more advanced disease extending into neurovascular bundle or seminal vesicles, results in potentially toxic doses being delivered to bladder and rectum. Hence, a stricter balance between delivery of optimum BED and limiting normal tissue toxicity needs to be attained. We irradiated the pelvic nodal region in all cases, based on the RTOG 9413 trial that demonstrated the benefit of this approach for high-risk PC [19]. Doses ≥ 60 Gy equivalent have been shown to be sufficient for gross nodal disease control [20]. Wang et al. [21] determined that a median SBRT dose of 37.4 Gy in four fractions to the rectum (BED = 154 Gy; α/β = 3) was associated with lesser late GI toxicity. Our treatment schedule complies with the doses and limits set by the above authors. The compliance to HT in terms of appropriate duration as per risk-category was 86.4%. Two out of the four patients who progressed had interrupted HT due to treatment-related side-effects. With this approach, we obtained a five-year bPFS of 91.4%, OS of 96.9% and ≥ Grade 3 late toxicity of 4.5% at a median follow-up of 63.5 months, which are better than other EH series utilizing a similar treatment approach (Table 3).

**Table 3:** Outcomes with EH boost for High-Risk Advanced PC SBRT: Stereotactic Body Radiation Therapy, IMRT: Intensity Modulated Radiation Therapy, 3DCRT: 3-dimensional Conformal Radiation Therapy, VMAT: Volumetric Modulated Arc Therapy, EH: Extreme Hypofractionation, Ac: Acute

Author	n	Stage / Risk	Fractionation, HT use	Median FU	Survival	≥ Gr 3 Toxicity
Miralbell [5]	50	Localised - 30, Advanced - 20 Low – 5, Int - 12, High - 33	Prostate RT 64-64.4 Gy in 32-35 fr (3DCRT / IMRT / VMAT), followed by EH boost 10-16 Gy in 2 fr (IMRT) to dominant tumour nodule HT – 33 pts	63 months	5-yr bPFS 98%	GU : Ac 4%, Late 0% GI : Ac 0%, Late 10%
Lin [6]	41	Localised – 24, Advanced - 17 High – 32, V. High – 9	Pelvic RT 45 Gy in 25 fr + EH boost to prostate 21 Gy in 3 fr HT – 38 pts	42 months	4-yr bPFS 91.9% 4-yr OS 92.2%	GU : Ac 0%, Late 0% GI : Ac 0%, Late 0%
Freeman [7]	1743	Localised & Advanced Low – 819, Int – 619, High – 168, V. High – 4 Metastatic – 3 Unspecified - 130	35-40 Gy in 5fr (Monotherapy) – 1499 pts Pelvic RT 45-50 Gy + Boost 19.5-21.75 Gy in 3 fr – 139 pts	24 months	2 yr bPFS 92% Low - 99% , Int (GS 3+4) – 97% Int (GS 4+3) – 85% High – 87%	GU : Late : 0% GI : Late : 0.1%
Anwar [8]	48	Localized - 28, Advanced - 20 Int – 14, High - 34	Pelvic RT 45 Gy in 25 fr + SBRT boost 17-21 Gy in 2fr HT – 45 pts	42.7 months	5-yr PFS 83% 5-yr bPFS 90%	GU: Ac:0%, Late: 2.1% GI : Ac : 0%, Late : 0%
Mercado [9] Paydar [10]	108	Localised – 107, Advanced - 1 Low – 4, Int – 45. High - 59	SBRT 19.5 Gy in 3 fr, f/b Pelvic IMRT 45-50.4 Gy in 25-28 fr HT – 70 pts	53 months	3-yr bPFS 100% 3-yr bPFS 89.8%	GU : Ac : 1%, Late : 6.5% GI : Ac : 0%, Late : 1%
Kim [11]	39	Localised – 35, Advanced - 4 Int – 20, High - 19	Pelvic RT 45 Gy in 25 fr + SBRT boost 21 Gy in 3 fr No HT	53.6 months	5-yr bPFS 94.7%	GU : Ac : 0%, Late : 0% GI : Ac : 0%, Late : 0%
Pryor (PROMETHEUS Trial) [12]	135	Localised & Advanced Int – 103, High - 32	SBRT 19-20 Gy in 2 fr, f/b IMRT 46 Gy in 23 fr or 36 Gy in 12 fr HT – 73 pts	24 months	2-yr bPFS 98.6%	GU : Ac : 0%, Late : 2.2% GI : Ac : 0%, Late : 2%
This report	44	All Advanced : High – 11 , V. High – 22, N1 – 9, M1 – 2	VMAT 50 Gy, f/b CK SBRT 18 Gy in 3 fr HT – All pts	63.5 months	5-yr PFS 88.2% 5-yr bPFS 91.4% 5-yr OS 96.9%	GU : Ac 2.3%, Late 4.5% GI : Ac 2.3%, Late 0%

Lin et al. [6] treated 41 high-very high-risk cases, of which 17 (41%) were advanced PC, with pelvic RT and EH boost of 21 Gy in three fractions to the prostate. At a median follow-up of 42 months, they reported a four-year bPFS of 91.9% and OS of 92.2%. In the registry of 743 patients by Freeman et al. [7], 175 (24%) belonged to high-very high and oligometastatic category. The combination of pelvic RT, along with prostate boost 19.5 to 21.75 Gy in three fractions was utilized in 139 (19%) patients. A two-year bPFS of 87% was reported. Anwar et al. [8] treated 48 patients, of which 34 (71%) were high-risk and 20 (42%) were advanced PC, with pelvic RT and SBRT boost of 17-21 Gy in two fractions to the prostate. At a median follow-up of 42.7 months, they reported a five-year bPFS of 90% and five-year PFS of 83%. In the PROMETHEUS trial [12], 135 localized and advanced PC cases, including 32 (24%) high-risk patients, were treated with SBRT 19-20 Gy in two fractions to prostate, followed by pelvic IMRT 46 Gy in 23 fractions or 36 Gy in 12 fractions. HT was used in 73 patients. A two-year bPFS of 98.6% was reported.

Among studies employing EH alone for advanced PC, Murthy et al. [22] treated 68 high-very high risk and node-positive cases, of which 50 (74%) were advanced PC, using tomotherapy-based IMRT to doses of 35-37.5 Gy to prostate and 25 Gy to nodes in five fractions, along with HT for at least two years in all patients. They reported a combined 1.5-year bPFS of 94% and OS of 97%. Although this study meets the radiobiological doses and limits criteria described above, the authors need to report much longer follow-up data to be of relevance. The HYPO-RT PC trial [23] included 1180 intermediate- and high-risk cases, of which 51 (4%) were advanced PC, and similar five-year bPFS of 84 % were obtained in both arms of EH 42.7 Gy in seven fractions and CF 78 Gy in 39 fractions. The proportion of advanced PC cases was severely limited in this trial. Despite this, our five-year outcomes are clearly superior to those reported in this trial.

Most series of advanced PC patients with reported median follow-up duration >four years have late ≥ Grade 3 GU and GI toxicity rates up to 6.5% and 3.3%, respectively [10-11]. Our toxicity rates are comparable to these. Efficient RT planning, use of dedicated rectal emptying and bladder filling protocols, omission of uninvolved portion of the seminal vesicles from the target volume, limiting of posterior PTV margins to limit the rectal volume being irradiated, delineation of urethra and limiting hot-spots inside it, daily image-guidance for VMAT, intrafraction motion tracking on CK by use of implanted fiducials, alternate-day treatment schedule for CK-SBRT, and delivering CK-SBRT before VMAT are some of the factors responsible for limiting late toxicity. A study by Feng et al. [24] compared late GU toxicity between two schedules of SBRT alone versus a combination of IMRT and SBRT boost similar to our study and found lesser toxicity with the latter approach. Patients with prostate volume >50cc at baseline have been shown to experience greater GU toxicity [25]. In our study, the median prostate volume was 43.9 cc before neoadjuvant HT and 23.5 cc before RT. Two of our patients (4.5%) developed urethral strictures. This agrees with the results by Awad et al. [26], who demonstrated pooled estimate prevalence of urethral strictures of 1.5% for external beam RT alone, 1.9% for brachytherapy alone, and 4.9% for a combination of both.

Efforts to further improve outcomes using different approaches for advanced and oligometastatic PC are ongoing. SBRT dose escalation to the dominant intraprostatic nodule as seen on MRI or PSMA-PET has been reported [5], with good short-term control rates. The NINJA (TROG 18.01) [27] is an extension of the PROMETHEUS trial [12] and compares SBRT boost 20 Gy in two fractions followed by pelvic RT 36 Gy in 12 fractions to SBRT 36.25 Gy (PTV) and 40 Gy (CTV) in five fractions. The PBS trial [28] is investigating toxicity and quality-of-life in advanced PC patients treated with pelvic RT 45-46 Gy followed by boost 32-33 Gy with CF or 19-21 Gy in three fractions with SBRT. The PRIME trial [29] compares EH 36.25 Gy in five fractions over seven to 10 days against the standard MH 68 Gy in 25 fractions over five weeks for high-very high and node-positive PC. Results of these trials shall help determine the most suitable hypofractionated schedule for advanced PC.

The idea of irradiating the primary tumour and the oligometastatic disease sites has been a subject of randomized trials in recent years. The STAMPEDE trial [30] randomized metastatic PC patients to standard of care HT or chemotherapy arms versus the experimental arm additionally incorporating RT to the prostate. The RT was either an MH schedule of 55 Gy in 20 fractions or an EH schedule of 36 Gy in six fractions, once-a-week delivery. Although there was no improvement in OS, the RT arm showed a statistically significant improvement in failure-free survival (HR 0.76, 95% CI 0.68-0.84; p < 0.0001). Results of the ongoing ORIOLE, FORCE, and PEACE1 trials shall further clarify the role of RT in oligometastatic PC.

Despite the good five-year outcomes, we do admit that our study has limitations of non-randomized design, relatively smaller patient numbers and follow-up duration. Since long-term tumour control is a combined effect of HT and RT, quantifying the individual contribution of RT dose-escalation by means of EH may be difficult. We do not believe that sequencing of the EH boost may alter disease outcomes, since the total BED is similar. The overall treatment time (OTT) does differ, being eight weeks in patients who received EH boost after the CF course, and six weeks in those who received it prior. This way, the reverse boost approach offers twin benefits of EH as well as reduction in OTT as compared to conventional and sequential boost regimens. As such, our patient numbers are too small to detect any difference in outcomes based on the differences in OTT, if any. It is worthwhile to try an appropriately adjusted combination of MH and EH for abbreviating the OTT further for this specific cohort of advanced and metastatic PC.

## Conclusions

Our phase I/II study proves that utilizing EH as a component of treatment for locally advanced, node-positive and oligometastatic PC, along with appropriate duration HT, can help attain excellent five-year control and survival rates, along with acceptable toxicity. This merits testing EH against a CF or MH treatment arm in a Phase III randomized trial. The anatomical location of the prostate and the biology of prostate cancer places it in a favorable situation where EH schedules are likely to enhance the therapeutic ratio further. With an increasing number of ongoing trials focusing on radical local treatment strategies for oligometastatic disease, the future appears bright for extending the domain of RT in this disease setting. Results of these trials shall help determine the best hypofractionated schedule for advanced and oligometastatic PC.
